# Causes and Consequences of Schadenfreude and Sympathy: A Developmental Analysis

**DOI:** 10.1371/journal.pone.0137669

**Published:** 2015-10-01

**Authors:** Rose Schindler, André Körner, Sylvia Bauer, Sarina Hadji, Udo Rudolph

**Affiliations:** Department of Psychology, Technische Universität Chemnitz, Chemnitz, Germany; University of Akron, UNITED STATES

## Abstract

Moral judgments and moral emotions are a ubiquitous feature of social interactions. Humans decide quickly and intuitively whether an action is morally right or wrong. Schadenfreude and sympathy, as emotional reactions to the misfortunes of others, are prototypical moral emotions. So far, however, little evidence exists concerning children’s understanding of schadenfreude. Within three studies, we investigated the experience of schadenfreude and sympathy among *N* = 364 children of different age groups. We interviewed the children while showing them picture stories. In the picture stories, we varied the behavior of the protagonist prior to a misfortune: (1) whether his behavior had been morally right or wrong, (2) whether the protagonist attained his goal, (3) whether the protagonist was responsible for the misfortune. In addition, in one study we varied (4) the emotional relationship of the interviewed children to the protagonist. Furthermore, we asked the children to decide whether they want to sit next to the protagonist or do him a favor. Results show that children experience sympathy as well as schadenfreude at the age of 4 years. Sympathy is more likely to arise when the protagonists of a story are likable, when these actors typically pursue morally positive goals, and if they are not responsible for their misfortune. In contrast, schadenfreude is more likely when the protagonist is disliked, when actors pursue immoral goals and if they are responsible for their misfortune. In addition, sympathy increases approach (helping behavior, sitting next to the agent and doing favors), whereas schadenfreude increases avoidance tendencies.

## Introduction

### Schadenfreude and Sympathy

Imagine two children on a snowy day playing in the park. Suddenly one of them stumbles and falls flat on his face into the snow. Close to tears, he struggles to his feet. How does the other child who observed the unlucky fellow react, with schadenfreude or with sympathy?

Schadenfreude and sympathy are two sides of the same coin—they are elicited in situations in which we observe another person who experiences a misfortune. In the case of schadenfreude, we feel pleasure in the other person’s misfortune (positive hedonic quality), while in the case of sympathy, we feel the pain of the other person (negative hedonic quality). Previous research focused primarily on the antecedents and consequences of sympathy and schadenfreude in adults, but much less research has examined these emotions in children. Nevertheless, the development of sympathy in children is rather well investigated; in contrast, little is known about the development of schadenfreude.

The goal of the present paper is to analyze the antecedents and consequences of sympathy and schadenfreude in children. We conducted 4 studies with *N* = 364 children between the ages of 3 and 8. In a pilot study, we interviewed parents and caregivers regarding their first recognition of schadenfreude in their children. Furthermore, we examined how often since then and in which settings the children display schadenfreude. In Study 1, we explore the impact of goal attainment prior to a misfortune on schadenfreude, sympathy, helping behavior and approach. In Study 2, we examine the influence of the personal relationship with the unlucky person on schadenfreude, sympathy and doing favors. Finally, in Study 3, we focus on the valence of a behavior prior to a misfortune and the responsibility for one’s misfortune on schadenfreude, sympathy, helping behavior and doing favors.

### Regulatory Functions of Schadenfreude and Sympathy

In an everyday situation like our example, the situational circumstances that cause one of the children to stumble can be diverse. The child could have fallen because he was reckless. In this case, displaying schadenfreude might be a signal to be more careful next time. On the other hand, the child might have stumbled because a third person pushed him. In this case, showing sympathy would be a comforting signal conveying that the accident was not his fault. In this case, Schadenfreude functions as a “stop” signal in social regulation processes indicating a prior misconduct or inattention of the actor [1]. Sympathy on the other hand is a moral “go” signal for the actor as it shows that he was not responsible for his misfortune [2]. Situations like the one in our example occur almost every day; therefore, both emotional reactions are salient in social life and help us to regulate our social interactions in terms of future behavior. As such, it is necessary to discover the basic principles underlying these emotion-regulatory processes.

### Predictors of Schadenfreude and Sympathy

As pointed out, another person’s misfortune is a prerequisite for both emotions. Previous research with adults has identified certain predictors that serve as additional preconditions for schadenfreude. For instance, deservingness of the misfortune is known to reliably elicit schadenfreude [3–6]. For example, van Dijk, Ouwerkerk, Goslinga, and Nieweg [7] found that the background of a negative event is an important predictor of feelings of schadenfreude.

Another predictor is the presence of a *comparative situation* that is related to one’s own feelings of inferiority towards the actor. In this way, schadenfreude is also linked to feelings of envy. This relation is particularly strong for interactions with close actors [8]. In addition, the *severity of the misfortune* must not be too intense. Imagine if the boy in the park passed out, because his fall was severe. Even if we feel schadenfreude for a second, this will turn into sorrow and fear, which then results in prompt helping behavior. Finally, *negative feelings* like envy, anger, rage, and hatred towards the actor will enhance feelings of schadenfreude [9]. This is associated with the first argument, as we are more likely to assume that a disliked person has greater deservingness of misfortune. We may think, for instance, that the other person’s experience of the negative event is justice being done [3].

Sympathy and schadenfreude have similar triggers. Similarly, *deservingness* is a crucial dimension. In contrast to schadenfreude, feelings of sympathy are more likely to be elicited when a misfortune is undeserved. In terms of attribution research, this clearly leads to the causal dimension of *controllability*. Rudolph, Roesch, Greitemeyer, and Weiner [10] showed in a meta-analysis that sympathy only arises when the actor has no personal responsibility for the misfortune. Rudolph, Schulz, and Tscharaktschiew [11] identify another prerequisite for sympathy, when they consider the morality of the actor’s behavior (“*ought*”). Thus, there is a focus on the moral value of the action that directly leads to the negative event. Accordingly, we show sympathy when a positive action fails, whereas sympathy is absent when we fail to achieve an immoral goal. Another factor associated with sympathy is whether the person experiencing the negative event is *liked or disliked*. This factor references very early cognitive psychological concepts [12].

### Consequences of Sympathy and Schadenfreude

Consequences of sympathy and schadenfreude can be broadly divided into approach or avoidance reactions. Sympathy elicits prosocial behaviors such as helping and discourages antisocial behaviors such as aggression [13–15]. Whereas other “stop” signal emotions like rage or anger elicit aggressive reactions towards the actor, the communication of schadenfreude is itself a punishment for the actor.

### Development of Schadenfreude and Sympathy

Prior research on children’s emotional development has largely focused on empathy (to feel as the other) and sympathy (to feel concern for the other). Even infants show reactions to the crying or distress of others [16]. Beginning at the age of two years, children empathize with others [17]. This ability is a prerequisite for perspective taking and thus for sympathy. Furthermore, three-year-old children begin to attribute causality to events and assume what other people feel, think, intend and expect [18,19]. This development results in the ability to feel and display sympathy.

As discussed above, schadenfreude can be seen as a “stop” signal and as a response to wrongdoing or carelessness. Children need to have a rudimentary understanding of the moral norms of society (e.g. prohibitions against hurting others or stealing things) and be able to perceive norm violations. Children between two and a half and three years old understand norms and recognize their transgression [20,21]. Furthermore, children between five and nine years old learn that the transgression of a norm will lead to immediate punishment. Young children see punishment as a deterrent to further wrongdoing and the stricter it is the more effective they think it will be [22]. In a first study of schadenfreude in preschool children, Schulz, Rudolph, Tscharaktschiew, and Rudolph [23] document that children feel and display schadenfreude beginning at age four. In this study, children were interviewed about their emotional and behavioral reactions towards the protagonist in a picture story. In these stories, the protagonist pursued a moral versus an immoral goal before experiencing a misfortune. Children were more likely to display schadenfreude when the protagonist of the picture stories pursued an immoral goal relative to a moral goal. In contrast, children were more likely to display sympathy and helping behavior when the protagonist pursued a moral goal.

Based on these initial results, we aim to advance our understanding of the causes and consequences of schadenfreude and sympathy. Specifically, we analyze the influences of goal attainment, personal relationship, valence of a behavior, and responsibility on schadenfreude and sympathy, as well as the consequences of these emotions on approach and avoidance behavior. In conclusion, we identified several antecedents and consequences of schadenfreude and sympathy.

### Ethical Aspects

The ethics committee of the Technische Universität Chemnitz approved the studies presented here (1) V-016-15-SM-UR-Schadenfreude-13122012 (13.12.2012) and (2) V-040-15-SM-UR-Schadenfreude-28112013 (28.11.2013).

## Pilot Study: Perception of Children’s Schadenfreude by Parents and Kindergarten Caregivers

We interviewed parents and kindergarten caregivers to explore their impressions. We assessed (1) whether parents and caregivers noticed schadenfreude in preschool children, (2) at what age they estimate that their children first experienced schadenfreude and (3) in which situations they noticed schadenfreude. We promoted the study to managers, caregivers and parents at six kindergartens in personal meetings and with informational leaflets. In a first step, parents gave their written consent for *N* = 120 children (age ranged from 3–7 years, Mage = 5.60 years, SD = 0.90 years; 55 girls, 65 boys) to participate in the study. The parents then completed an anonymized and brief questionnaire. In a second step, caregivers received corresponding questionnaires for the 120 children and returned completed questionnaires for *N* = 108 children.

### Parents

The parent questionnaire included three questions: (1) Has your child ever felt schadenfreude? (*yes/no*), (2) If so, how old was your child, when he/she felt it for the first time? (*2 Years*, *2½ years*, *3 years*, *3½ years*, *4 years*, *4½ years*, *5 years*), and (3) Can you briefly describe a situation in which you were able to observe your child experiencing schadenfreude? Out of the 120 parents, 80 (66%) indicated that their child had already experienced schadenfreude; whereas 35 parents (30%) claimed to never have seen schadenfreude in their child (5 parents (4%) provided no response). Out of those 116 parents who noticed schadenfreude, 69 estimated the age of their child. Of these, only three parents (4%) estimated the onset of schadenfreude around the age of two and a half. Of the remaining parents, 15 parents (22%) estimated their children to be three years old, 11 parents (16%) estimated their children to be three and a half years old, 21 parents (30%) estimated their children to be four years old, 11 parents (16%) estimated their children to be four and a half years old, and 8 parents (12%) estimated their children to be five and a half years old. Five parents could not remember a specific age and 42 parents did not provide a response. Most of these parents estimate that their children were between three and four years old at the first occurrence of schadenfreude, this corresponds with results of Schulz et al. [23].

### Caregivers

The caregiver questionnaire contained three questions: (1) Did this child ever feel schadenfreude? (*yes/no*), (2) If so, how old was this child, when he/she felt it for the first time? (*2 years*, *2½ years*, *3 years*, *3½ years*, *4 years*, *4½ years*, *5 years*) and (3) Can you briefly describe a situation in which you were able to observe your child experiencing schadenfreude? The caregiver observed schadenfreude in 59 (55%) of the 108 children. For 46 of the 59 children, caregivers estimated the age at the first occurrence of schadenfreude. The caregivers estimated one child (2%) at the age of two years and two children (4%) at the age of two and a half years. In addition, the caregivers estimated that 14 children (30%) first experienced schadenfreude at the age of three years, 4 children (9%) at the age of three and a half years, 12 children (27%) at the age of four years, six children (13%) at the age of four and a half years, and seven children (15%) at the age of five years.

Both the parents and caregivers reported that more than half of the children had experienced schadenfreude. The parents’ and caregivers’ assessment of the children’s age at the first occurrence of schadenfreude is consistent. According to them, children first exhibit schadenfreude between the third and fourth year of their life. This is consistent with the results of Schulz et al. [23] and supports our assumption that schadenfreude is a basic emotion that is learned quite early.

### Typical Situations

Parents and caregivers reported many situations eliciting the emotion of schadenfreude. This also indicates that schadenfreude is a common emotion in children’s everyday lives. We categorize the stories mainly into three prototypical situations triggering schadenfreude: (1) misfortunes of parents or siblings (in which something fell down; someone trips or misspeaks), (2) competitive situations with peers in sports or social games (in which the gloating child has higher skills or cheats) and (3) situations in which the gloating child did not get in trouble but his/her peers did (e.g. were yelled at).

### Design of Material Used in the following Studies

Based on the situations described in the pilot study, we designed twenty picture stories. To ensure that this material reliably triggers schadenfreude, we interviewed students and then children to ensure that our stories reliably elicited schadenfreude. Specifically, we read the stories to the students and children in a randomized order and asked them to evaluate them for comprehensibility. In addition, participants indicated how much the stories triggered schadenfreude, sympathy and help behavior and how severe the accident was. Out of the twenty designed stories, we selected the five with the highest comprehensibility that triggered strong emotions. These were used in the following studies.

## Study 1: Goal Attainment and Approach

In Study 1, we analyze whether schadenfreude and sympathy are influenced by the attainment of an immoral goal. Imagine the child who stumbled was about to throw a snowball. Maybe he throws the snowball and hits the other child in the snowy park before falling into the snow or in contrast, he falls before he can throw the ball. In which of the two possibilities would another person feel more schadenfreude? In this study, the goal of our protagonist is always negative. When a person has an accident while pursuing a negative goal, the misfortune is often viewed with satisfaction as a "deserved punishment" for doing something wrong (see [24]). Thereby, the attainment of the goal as well as the intention to attain a negative goal are sufficient to elicit negative emotions at least in adults [11]. We expect that children are more likely to experience schadenfreude when the protagonist suffers a misfortune after attaining a negative goal than in situations in which the protagonist does not attain the negative goal. In addition, we predict that children are more likely to experience sympathy and provide help if the child does not attain his goal before the misfortune occurs. Furthermore, we examine the subsequent behavioral reaction of an observer who felt schadenfreude or sympathy. Specifically, we compare the likelihood of approach and avoidance behavior of an observer towards the target of his emotional reaction.

### Method

#### Participants

We promoted study participation to kindergartens and parents at a parents' evening, prepared leaflets, and asked for written consent. Based on the written consents, we interviewed *N* = 201 preschooler’s. We excluded six children from data analyses due to missing answers and poor understanding of the stories. The final sample included *N* = 195 children (87 girls, 108 boys) with a mean age of *M* = 64.85 months, *SD* = 9.74. We divided the children into three age groups for some of the following analysis: 36% were between four and five years old (*M* = 53.93 months, *SD* = 3.49; 31 girls, 39 boys), 33% were between five and six years old (*M* = 65.89 months, *SD* = 3.33; 40 girls, 25 boys) and 31% were aged six years and above (*M* = 76.45 months, *SD* = 2.69; 16 girls, 44 boys).

#### Experimental Design

We designed a scenario-based interview consisting of pictures stories. We asked the children to imagine being the observer of the described events. Using a 2 x 2 within-subjects design, we systematically varied the gender of the protagonist (*male* vs. *female*) and goal attainment (*attained* vs. *not attained*) of an action before the misfortune happened. This resulted in four stories: One with a female and one with a male protagonist who respectively attained or failed to attain an immoral goal. We designed the stories and pictures carefully to control for potentially confounding factors. Thus, we counterbalanced protagonist's gender with the gender of the victim (male protagonist female victim as well as female protagonist and male victim). Moreover, the quality of their relationship was also held constant since protagonist and victim were always siblings.

#### Material and Procedure

**Fig 1:**
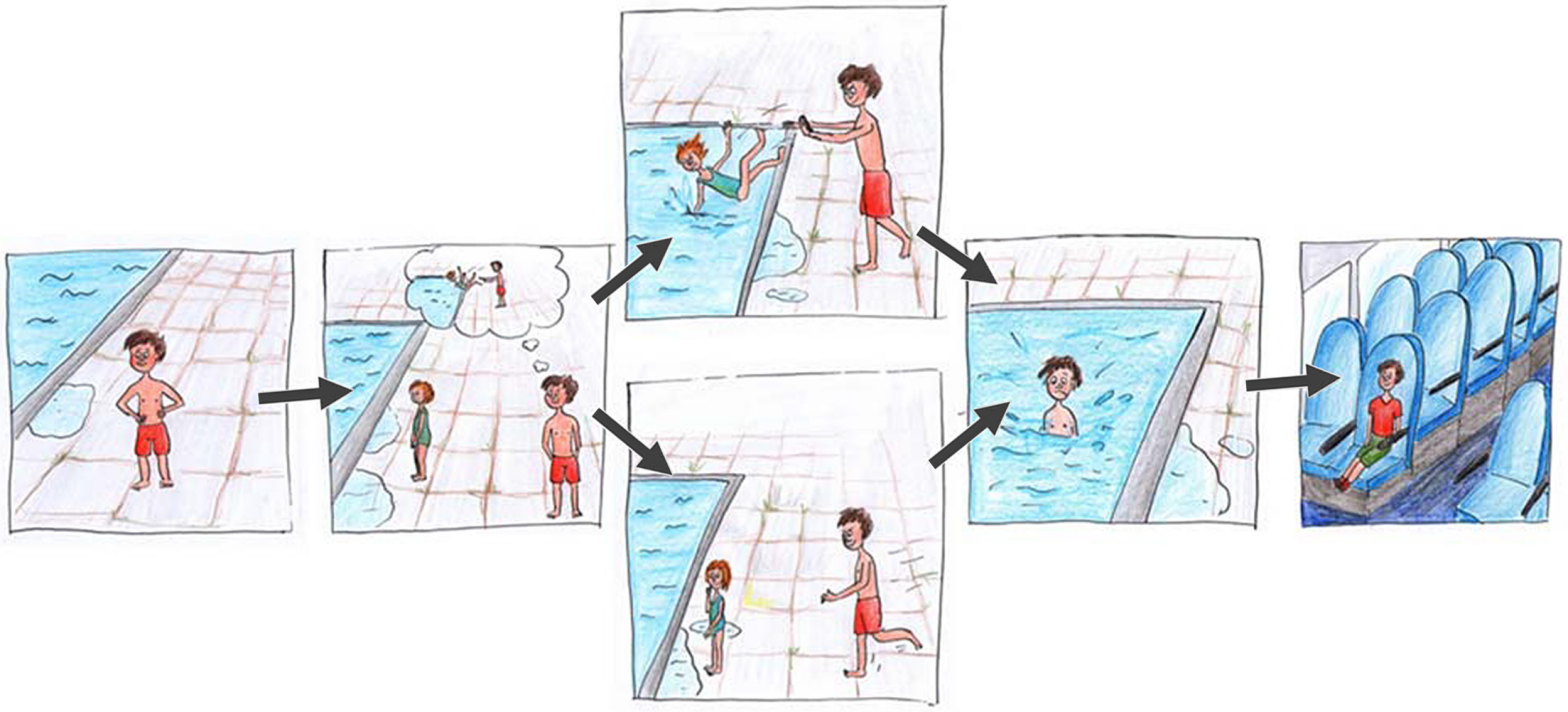
Example of a picture story used in Study 1: “Max and the swimming pool”.

Two female interviewers, who were about the same age, undergraduate students (psychology) and had both full knowledge of study hypotheses, tested the children. One of two female interviewers (person A or B) tested the children individually in a quiet room at the kindergarten. Each individual session took approximately 20 minutes. The interviewer read two stories aloud to the child while simultaneously showing a sequence of five different pictures (see Fig 1). In the beginning, a protagonist (main character) was introduced. His or her goals and actions were described, which always involved an interaction partner. At the end of the story, the protagonist suffered a misfortune. Regarding independent variables, we manipulated two pictures within the stories. For example, the scenario “Sarah and the plum tree” was about a girl named “Sarah” climbing a tree in order to pick plums to throw them at her brother. She either attained the goal by throwing the plums at her brother and subsequently fell from the tree or fell before attaining her goal. In the scenario “Max and the swimming pool”, the protagonist “Max” wants to nudge his sister into the swimming pool. Either he attained his goal before falling into the pool as well or he slipped and fell before nudging his sister into the water. Both stories ended with a picture presenting either “Sarah” or “Max” sitting in a Bus.

Afterwards, the children answered (in a random order) how much they experienced sympathy and schadenfreude towards the protagonist, since the protagonist of the stories is always the target of the emotions. Furthermore, the children indicated how likely it is that they would help the protagonist. In addition, they evaluated the severity of the misfortune that happened to the protagonist and the willingness to sit next to the protagonist in the bus. The children answered by using a 5-point rating scale (0 = *not at all*, 4 = *very much*). The rating scale was displayed as a triangle in which children pointed to the section that best matched the intensity of their feelings. We trained participants on the use of the five-point scale by rating some of the children’s favorite and least favorite foods. Finally, we rewarded the children for their participation given that the kindergarten teacher allowed this (e.g., playing a game with the interviewer, getting some sweets). After each interview, the interviewer assessed the children’s performance with respect to their understanding of the stories, their comprehension of the scale, attention and motivation by using a 5-point rating scale (0 = *very poor*, 4 = *very good*).

### Results

In this study, we tested the influences of (1) goal attainment and (2) gender of the protagonist on the dependent variables by using regression analyses. Therefore, we conducted within subjects’ analyses. To control for confounding variables, we also included age and gender of the interviewed child, as well as that of the interviewer. For a clear interpretation of possible age effects, we also used contrast analyses.

Table 1 shows the mean values of the children’s emotional reactions (sympathy, schadenfreude), behavioral tendencies (helping behavior, approach), and the control variable (perceived severity of the misfortune) for three different age groups.

**Table 1:** Mean Values of Schadenfreude, Sympathy, Helping Behavior, Doing a Favor and Severity of a Misfortune.

	4 Years	5 Years	6 Years
	*M (SD)*	*M (SD)*	*M (SD)*
Schadenfreude	3.20 (1.71)	2.85 (1.78)	2.59 (1.62)
Sympathy	2.98 (1.53)	2.92 (1.58)	3.03 (1.56)
Helping behavior	3.00 (1.59)	3.29 (1.64)	3.18 (1.45)
Approach	3.33 (1.60)	3.21 (1.70)	3.58 (1.46)
Severity of the Misfortune	2.54 (1.55)	2.62 (1.61)	2.87 (1.46)

#### Control Variables

**Quality of participation:** Story comprehension (*M* = 3.31, *SD* = 0.77), appropriate use of the triangle rating scale (*M* = 2.91, *SD* = 0.81), attention and motivation (*M* = 3.38, *SD* = 71) were observed by the interviewers to be between satisfying and very good.

**Severity of misfortune:** Overall, children evaluated the severity of the misfortunes as moderate (*M* = 2.67, *SD* = 1.59). Notably, the regression analysis indicated that children’s evaluations are sensitive to the interviewer who presents the stories, β = .16, *p* < .01. This showed that the manner in which the interviewer told the stories influenced participants’ perceptions. None of the other factors had a significant effect.

#### Emotional Reactions

**Schadenfreude:** hildren reported higher degrees of schadenfreude when the protagonist attained an immoral goal and then suffered a misfortune, compared to an unlucky actor who did not attain this goal before the misfortune happened (see Table 2). Moreover, they reported less schadenfreude when the severity of a misfortune was perceived as high, compared to a scenario where a misfortune was perceived as less severe. Furthermore, the age of the interviewed children had a small effect. The contrast analysis revealed a significant linear effect, *F*(1, 390) = 24.13, *p* < .01, *r* ^*2*^ = .06, indicating that younger children reported more schadenfreude than older ones. Additionally, the interviewer had a significant effect on the evaluation, indicating that children reported higher degrees of schadenfreude by interviewer A compared to interviewer B. There were no effects for the gender of the protagonist and the gender of the interviewed children.

**Table 2:** Multiple Regression for Schadenfreude and Sympathy.

	Schadenfreude			Sympathy		
	*B*	*SE B*	β	*B*	*SE B*	β
Constant	1.77	0.63		0.80	0.56	
Gender of interviewed Children	0.15	0.17	0.04	-0.26	0.16	-0.08
Age	-0.28	0.10	-0.13^**^	-0.01	0.09	0.00
Interviewer	-0.54	0.17	-0.15^**^	-0.21	0.16	-0.07
Severity of Misfortune	-0.12	0.05	-0.11^*^	0.27	0.05	0.28^***^
Gender of Protagonist	-0.12	0.08	-0.07	0.18	0.08	0.11^*^
Goal Attainment	-0.18	0.08	-0.10^*^	0.05	0.08	0.03

*Notes*.

Schadenfreude *R* ^2^ = .08

Sympathy *R* ^2^ = .09

*p < .05

**p < .01

***p < .001.

**Sympathy:** Children did not differentiate between attaining a goal versus failing to attain a goal before suffering a misfortune. In contrast, we found a significant effect for the protagonists’ gender, reflecting that children felt more sympathy for “Sarah’s” misfortune than “Max’s” accident. In addition, there was a significant effect of the severity of the misfortune. Relative to “Max’s” accident, children evaluated “Sarah’s” accident as worse. There was no effect of the interviewer, gender or age of the interviewed children.

#### Behavioral Tendencies

**Helping behavior:** Children did not differentiate between the male and female protagonist “Max” and “Sarah” concerning their intention to help (see Table 3). Furthermore, there was no effect of goal attainment. However, there was a negative effect of the perceived severity of the misfortune on helping behavior. Indicating that children were more motivated to help given a severe misfortune compared to a scenario where the misfortune was perceived as less severe. According to the regression analysis, the gender of the interviewed children had a small effect on helping behavior. The results indicate a trend that girls intended to help more often than boys, *t*(1,388) = 1.91, *p* = .06, *d* = 0.19. There was also a significant interviewer effect, indicating that children reported more helping behavior by interviewer B compared to interviewer A.

**Table 3:** Multiple Regression for Helping Behavior and Approach.

	Helping Behavior			Approach		
	*B*	*SE B*	β	*B*	*SE B*	β
Constant	1.05	0.58		0.51	0.59	
Gender of interviewed Children	-0.40	0.16	-0.13*	-0.30	0.16	-0.09
Age	0.10	0.10	0.05	0.11	0.10	0.06
Interviewer	-0.51	0.16	-0.16**	-0.14	0.17	-0.04
Severity of Misfortune	0.13	0.05	0.13*	0.11	0.05	0.11*
Gender of Protagonist	0.06	0.08	0.04	0.05	0.08	0.03
Goal Attainment	0.11	0.08	0.07	0.17	0.08	0.11*

*Notes*.

Helping Behavior *R* ^2^ = .05

Approach R^2^ = .03

*p < .05

**p < .01.

**Approach:** Children chose more frequently to sit next to the protagonist, when “Sarah” and “Max” failed to attain an immoral goal before suffering a misfortune compared to a scenario where they attained an immoral goal. Furthermore, children chose more frequently the seat next to the protagonist when the misfortune was perceived as severe in contrast to a less severe misfortune. There was no effect of protagonist gender, interviewer, age and gender of the interviewed children.

**Fig 2:**
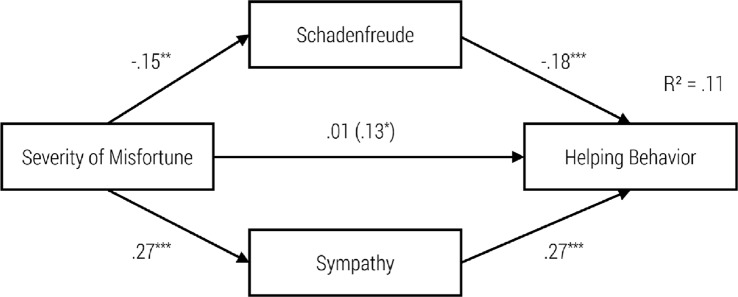
Path Model for the Impact of Severity of the Misfortune on Schadenfreude, Sympathy and Helping Behavior with standardized regression coefficients β and the Impact without Schadenfreude and Sympathy in Brackets, **p < .01; ***p < .001.

**Fig 3:**
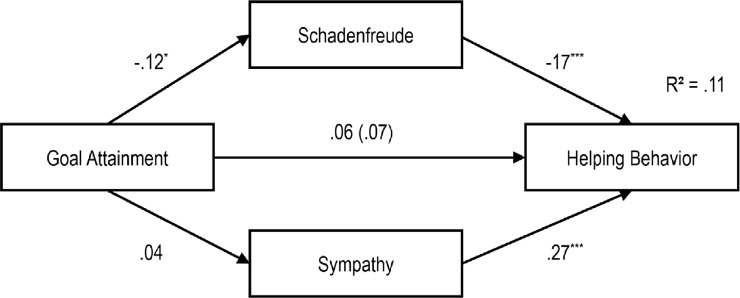
Path Model for Impact of Goal Attainment on Schadenfreude, Sympathy and Helping Behavior with standardized regression coefficients β and the Impact without Schadenfreude and Sympathy in Brackets, *p < .05; ***p < .001.

**Fig 4:**
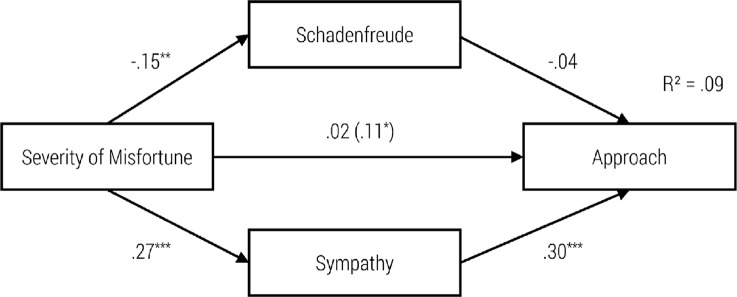
Path Model for Impact of Severity of the Misfortune on Schadenfreude, Sympathy and Approach with standardized regression coefficients β and the Impact without Schadenfreude and Sympathy in Brackets, **p < .01; ***p < .001.

**Fig 5:**
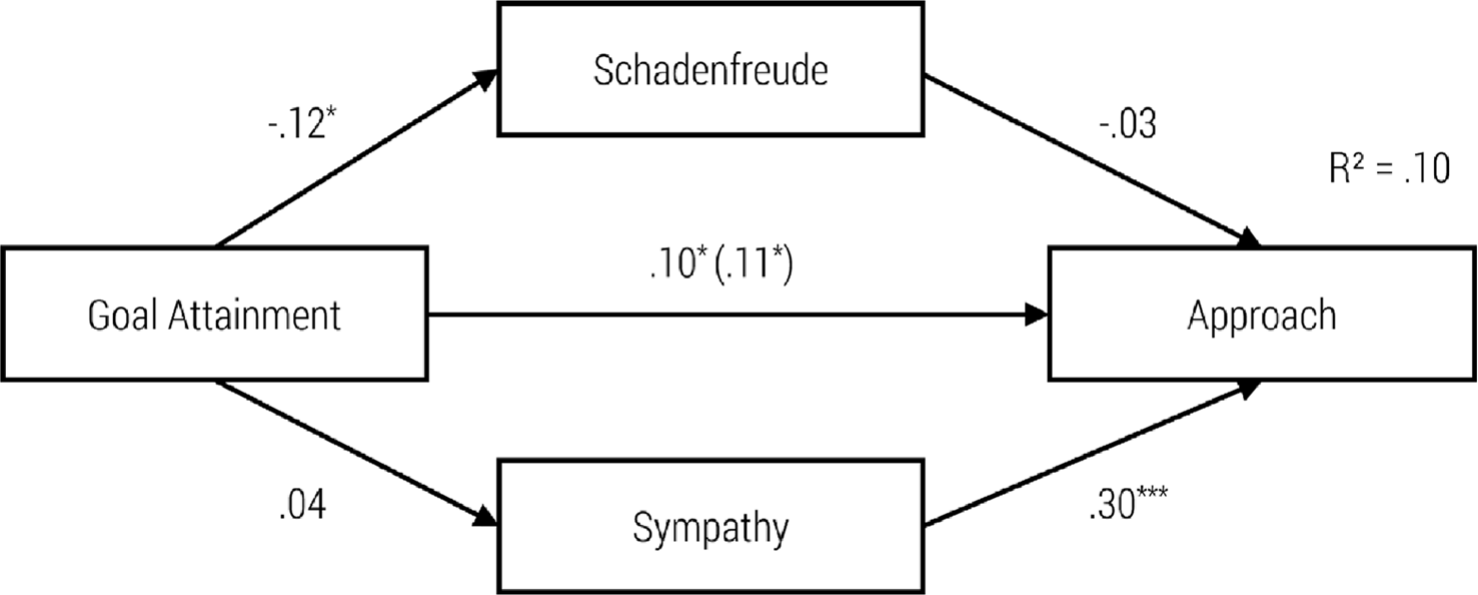
Path Model of Impact of Goal Attainment on Schadenfreude, Sympathy and Approach with standardized regression coefficients β and the Impact without Schadenfreude and Sympathy in Brackets, *p < .05; ***p < .001.

To illustrate the concrete impact of our independent variables on schadenfreude, sympathy, helping behavior and approach, we calculated four path models (see Figs 2–5).

As can be seen in the first model (Fig 2), the severity of the misfortune had a significant negative influence on schadenfreude, and schadenfreude had a significant negative influence on helping behavior. Furthermore, there was a significant positive relationship between severity of the misfortune and sympathy, as well as between sympathy and helping behavior. When adding the indirect paths from severity of misfortune to helping behavior via both emotions, then the standardized coefficient of the direct path decreased from *r* = .13 to *r* = .01. Hence, the model did confirm a total mediation. Testing the whole model according to the procedure proposed by Hayes [25] indicated significant indirect effects for schadenfreude (z = 2.223, p > .01) and sympathy (z = 3.830, p = .000).

In the second model (Fig 3), goal attainment had a significant negative effect on schadenfreude, and schadenfreude had a significant negative effect on helping behavior. Although goal attainment had no significant effect on sympathy, we found a significant positive effect from sympathy on the tendency to help. Moreover, goal attainment had no significant direct effect on helping behavior. In this sense, the requirements for mediation were not met. Hence, due to the non-significant direct and indirect paths the model did not confirm the mediation effect of schadenfreude and sympathy.

As can be seen in the third model (Fig 4), the severity of the misfortune had a significant negative effect on schadenfreude. Nevertheless, schadenfreude had no significant effect on approach. Furthermore, there was a significant positive relationship between severity of the misfortune and sympathy as well as between sympathy and approach. In addition, when adding the indirect paths then the severity of the misfortune had no significant direct influence on the choice of the seat. Again, due to the non-significant direct and indirect paths the model did not confirm the mediation effect of schadenfreude and sympathy.

In model 4 (Fig 5), goal attainment had a significant negative effect on schadenfreude. Nevertheless, schadenfreude had no significant effect on approach. Furthermore, goal attainment had no significant effect on sympathy. Nevertheless, sympathy had a significant positive effect on approach. Moreover, goal attainment had a direct significant effect on choice of seat. Nevertheless, due to the non-significant indirect paths the model did not confirm the mediation effect of schadenfreude and sympathy.

### Discussion

In this study, we focused on two research questions. First, we wanted to know whether one’s goal attainment of an immoral goal and the severity of a misfortune determines the experience of schadenfreude and sympathy. Furthermore, we hypothesized that these two emotions predict approach and prosocial behavior (i.e., helping behavior) towards the unfortunate protagonist.

#### Emotional Reactions

**Schadenfreude:** According to our results, the experience of schadenfreude is determined by goal attainment, severity of misfortune, the interviewer and age of the interviewed child. Specifically, the attainment of immoral goals increased the likelihood of schadenfreude relative to a failure to attain these goals. As described earlier, showing schadenfreude is a way to punish people for their wrongdoing. Thus, people are less likely to punish others if they failed to attain an immoral goal. As expected, schadenfreude was more likely to be elicited in situations in which the misfortune was perceived as less severe. This is consistent with previous research emphasizing the importance of low severity in triggering schadenfreude as opposed to sympathy [26]. Furthermore, the person who interviewed the children, or to be more precise, the way the children were interviewed had an effect. The children reported more schadenfreude in interviews with person A. This indicates that children can be easily influenced concerning revealing their schadenfreude, and highlights the need for interviewer training to ensure that interviewers behave as similarly as possible. In addition, younger (as compared to older) children reported more schadenfreude. Thus, children may learn to regulate their emotional expression according to societal rules or they start to gain a deeper social cognitive understanding. This may derive from children’s increasing awareness of others’ mental states e.g., when an observed child has an accident, it is already suffering and that is punishment enough. This refers to the phenomenon described as “happy victimizer” [27].

**Sympathy:** According to our results, sympathy is primarily determined by two factors: the gender of the protagonist/scenario and the severity of the misfortune. However, the relation between the gender of the protagonist and sympathy is not completely proven, because the gender of the protagonist is always linked to a different scenario and thus to a different type of accident. Thus, the finding might be due to the gender of the protagonist or the differences in the story. As expected, the severity of a misfortune is a particularly important predictor of an observer’s emotional reaction. Consequently, the more severe we judge an accident, the more sympathy we feel. Furthermore, children feel more sympathy for “Sarah” as compared to “Max”. This might be due to the fact that Max’ accident is evaluated as less severe than Sarah’s accident.

#### Behavioral Tendencies

**Helping behavior:** The desire to help another child is influenced by the interviewer, the severity of the protagonist’s misfortune and the gender of the interviewed child. Children report more helping behavior when interviewed by person B (see schadenfreude). Furthermore, to a smaller extend, helping behavior is also more likely when the accident is perceived as more severe. This is consistent with the results for sympathy. Since sympathy is a motivator for helping behavior, both are elicited by similar conditions [14]. In addition, our results suggest that girls intended to help more often than boys did. This is consistent with the social-role theory of gender and helping, as the female gender role fosters caring helping, rather than chivalrous and heroic helping [28].

**Approach:** As expected, goal attainment and the severity of misfortune affect tendencies to approach another person. Children tend to approach more when others fail to attain their negative goals. In contrast, they do not want to sit next to the protagonists of our stories when they achieved an immoral goal. In addition, children approach others more when the observed accidents are perceived as severe.

### Relationship between Triggering Factors, Moral Emotions and Behavioral Tendencies

The path models presented here integrate schadenfreude and sympathy as mediators of the relation between goal attainment and severity of the misfortune on one hand and approach behavior or helping behavior on the other hand. The severity of the misfortune has an emotionally mediated effect on helping, via schadenfreude as well as sympathy. High severity of the accident increases feelings of sympathy and decreases feelings of schadenfreude. Sympathy encourages helping whereas schadenfreude decreases the motivation to help.

Likewise, the severity of the misfortune has an indirect effect on the tendency to approach via sympathy. Higher perceived severity of a misfortune increases sympathy and encourages approach behavior (sitting next to the unlucky fellow). Furthermore, high-perceived severity of a misfortune decreases schadenfreude; however, schadenfreude has no significant (negative) effect on approach behavior.

In line with our expectations, goal attainment has an indirect effect on helping behavior via schadenfreude. The attainment of an immoral goal increases schadenfreude, which in turn decreases helping behavior. In contrast, goal attainment has no influence on sympathy. Nevertheless, the experience of sympathy increases the likelihood of helping behavior. On the other hand, the attainment of an immoral goal elicits schadenfreude, which has no influence on approach behavior. In contrast, goal attainment has no impact on sympathy, although sympathy predicts approach behavior (sitting next to a child on the bus).

## Study 2: Type of Relation and Granted Benefit

In Study 2, we analyzed the impact of the emotional relationship (*like* vs. *dislike*) with a protagonist (who pursues an immoral goal) on schadenfreude and sympathy. In contrast to Study 1, we attempt to assess actual prosocial behavior, because it may be more appropriate to access actual behavior by asking children to do something instead of the hypothetical question used before. Since this procedure is closer to the everyday life of the interviewed children, we expect a higher comprehension and response rate.

The notion that the quality of our relationship with another person predicts sympathy or schadenfreude is not new. In his early cognitive emotion theory, Alexius Meinong [12] assumed that like and dislike towards an actor predict our emotions in response to his misfortune. According to Meinong, schadenfreude is more likely when we dislike the suffering actor, whereas sympathy is more likely when our relation to the actor is positive.

The tendency to compensate someone in case of a misfortune is a common reaction. Thus, granting or withholding a favor are manifestations of approach and avoidance behavior. Doing someone a favor or providing material support is a more tangible than a mere intention to do something. The concept of gratification can be found in all kinds of cultural settings and even at early stages of development [29]. Distributing resources from one to another is a basic indicator of support [30] and follows norms and rules [31]. In addition, the exchange of food is a common behavior intended to overcome previous disadvantage [32]. Therefore, we decided to use gratification behavior as an indicator of approach and avoidance in response to a misfortune.

### Method

#### Participants

We examined first graders at an elementary school. In order to ensure participation, we explained the study design and discussed potential concerns with the teachers and caregivers as well as the parents. Prior to the study, we requested consent forms from all parents. Finally, *N* = 27 children (*M* = 91.26 months; 11 girls, 16 boys) took part in this study.

#### Material and Procedure

**Fig 6:**
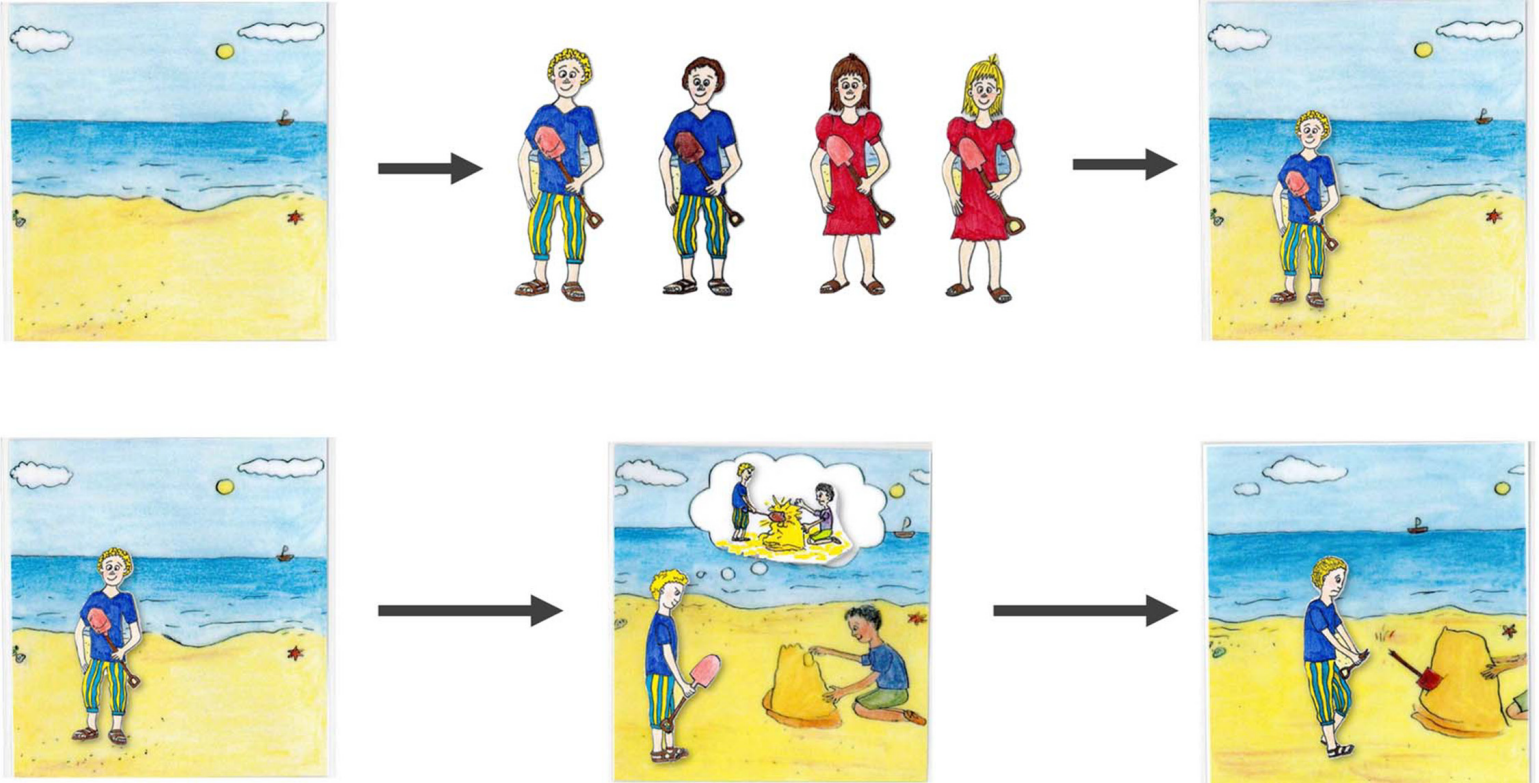
Example of a picture story used in Study 2: “sand castle” with four different characters *(above)* and a possible resulting picture story *(below)*.

One female interviewer, who was a graduate student (psychology) and had full knowledge of study hypotheses, tested the children. Prior to the study, we trained the interviewer by test interviews that were videotaped and subsequently analyzed. A female interviewer in a quiet room inside the school tested children individually. We used a scenario-based interview consisting of two picture stories that presented everyday life situations. In order to manipulate the children’s relationship to the specific protagonist within the stories, we asked them to name two classmates. One of these two classmates should be a classmate the children were least willing to have as a seatmate, i.e., someone they rather do not like that much. The other one should be their favorite seatmate, i.e., someone they like very much. We recorded the two names and asked the children to provide the two classmates’ hair color and gender. Then, we asked the participating children to imagine they were the observer of two stories. Next, we narrated the two picture stories “plum tree” and “sand castle” by placing a character, intended to look like the classmates that had been described earlier, into the three background pictures for each of the two stories. We used one of four already prepared characters, that is, either into the background stories (a light-haired or dark-haired boy or a light-haired or dark-haired girl. Fig 6 shows the backgrounds of the two stories as well as the characters we inserted. The sequence of stories (*plum tree* vs. *sand castle*) and the relationship to the protagonist (*like* vs. *dislike*) were fully randomized.

As in Study 1, the interviewer read the two stories to the child while at the same time showing the respective pictures. Both stories showed a protagonist who tried to attain an immoral (antisocial) goal that he or she was unable to accomplish because of a misfortune. The “plum tree” scenario described a child climbing on a tree in order to pick some plums and throw them at another child. The plan failed when the protagonist fell off the tree and sustained mild injuries. In the “sand castle” scenario, the protagonist was about to destroy another child’s sand castle with a spade. In the process of doing so, the beloved spade broke in two and the protagonist was sad.

Participants indicated on an 8-point rating scale (1 = *not at all*, 8 = *very much*) how much they would experience sympathy and schadenfreude. The children used a triangle to rate their feelings. We trained participants beforehand on how to use the eight-step scale by having them rate their favorite and least favorite foods. Finally, we also assessed the granting of a reward. The interviewer marked two additional little bags of sweets with the name of the two protagonists (relationship type: *positive* vs. *negative*). The children were then asked to decide whether these sweets should be granted to the protagonists or not. Furthermore, we informed the children that we would keep their choice a secret.

### Results

#### Emotional Reactions

**Schadenfreude:** Boys (*M* = 5.13, *SD* = 3.01) and girls (*M* = 5.14, *SD* = 3.00) did not differ significantly in their feelings of schadenfreude overall, *t*(52) = -.01, *p* **=** .99. Moreover, children reported more schadenfreude when they had a negative relationship with the story’s protagonist (*M* = 6.41, *SE* = 0.49) as compared to a positive relationship (*M* = 3.85, *SE* = 0.55); *t*(52) = 3.47, *p* **<** .001, effect-size *r* = .43. These findings are also supported by a regression analysis with schadenfreude as the outcome and age, gender, and relationship type with the protagonist as predictors. As shown in Table 4, age and gender did not predict schadenfreude. Relationship type was a significant negative predictor of children’s feelings of schadenfreude (β = -.44, *p* < .001).

**Table 4:** Multiple Regressions for Schadenfreude and Sympathy.

	Schadenfreude			Sympathy		
	*B*	*SE B*	β	*B*	*SE B*	β
Constant	-11.29	7.51		11.28	6.76	
Gender of Interviewed Children	-.10	.86	-.02	.57	.78	.09
Age	-.13	.82	.20	-.12	.07	-.20
Relationship with Protagonist	-1.50	.42	-.44***	1.43	.38	.46***

*Notes*.

Schadenfreude *R* ^2^ = .24

Sympathy *R* ^2^ = .26

***p < .001.

**Fig 7:**
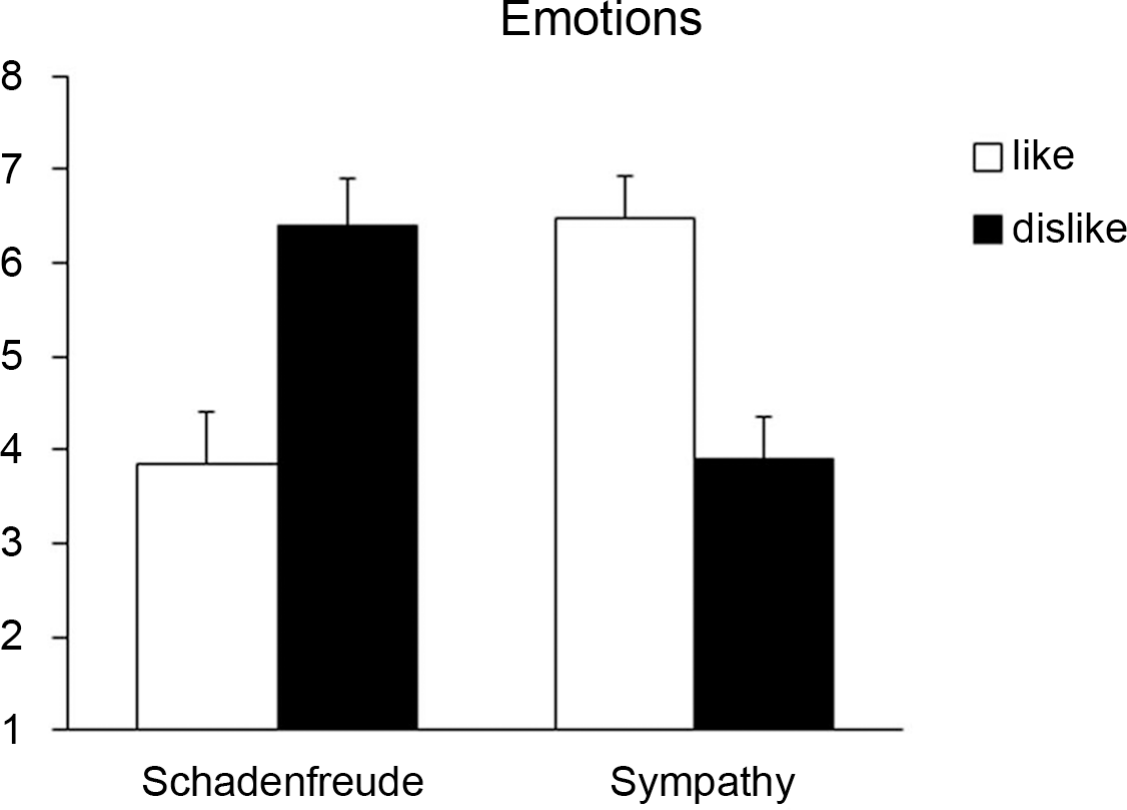
Children’s expression of Schadenfreude and Sympathy depending on like and dislike of the protagonist.

**Sympathy:** Boys (*M* = 5.03, *SD* = 2.90) and girls (*M* = 5.41, *SD* = 2.46) did not differ significantly in their feelings of sympathy, *t*(52) = —.50, *p* **=** .62. Children showed more sympathy when they had a positive relationship with the protagonist (*M* = 6.48, *SE* = 0.45) than a negative relationship (*M* = 3.89, *SE* = 0.47), *t*(52) = -3.97, *p* **<** .001, effect size *r* = .48. Fig 7 shows the means for schadenfreude and sympathy for relationship type (like vs. dislike).

Again, age and gender were not associated with the outcome, when examining a regression analysis with sympathy as the outcome and age, gender, and relationship type as predictors (see Table 4). Similar to schadenfreude, relationship type significantly predicted children’s feelings of sympathy (β = .46, *p* < .001). Thus, a positive relationship with the protagonist predicted higher feelings of sympathy.

#### Behavioral Measures

**Granting a reward:** When being asked to grant them a reward or not, children differentiated between a liked versus disliked protagonist. Nearly all children (26 out of 27) gave a bag of sweets to the liked protagonist, whereas only 11 out of 27 of the disliked protagonists were rewarded. This was a significant finding, *χ* ^*2*^(1) = 19.32, *p* < .001. Odds ratios showed that the children were over 37 times more likely to grant a reward to a liked protagonist than a disliked protagonist was. Moreover, schadenfreude was negatively related to the granting of a reward (*r* = .29, *p* = .03), while sympathy was positively associated with compensatory behavior (*r* = .41, *p* = .00).

**Fig 8:**
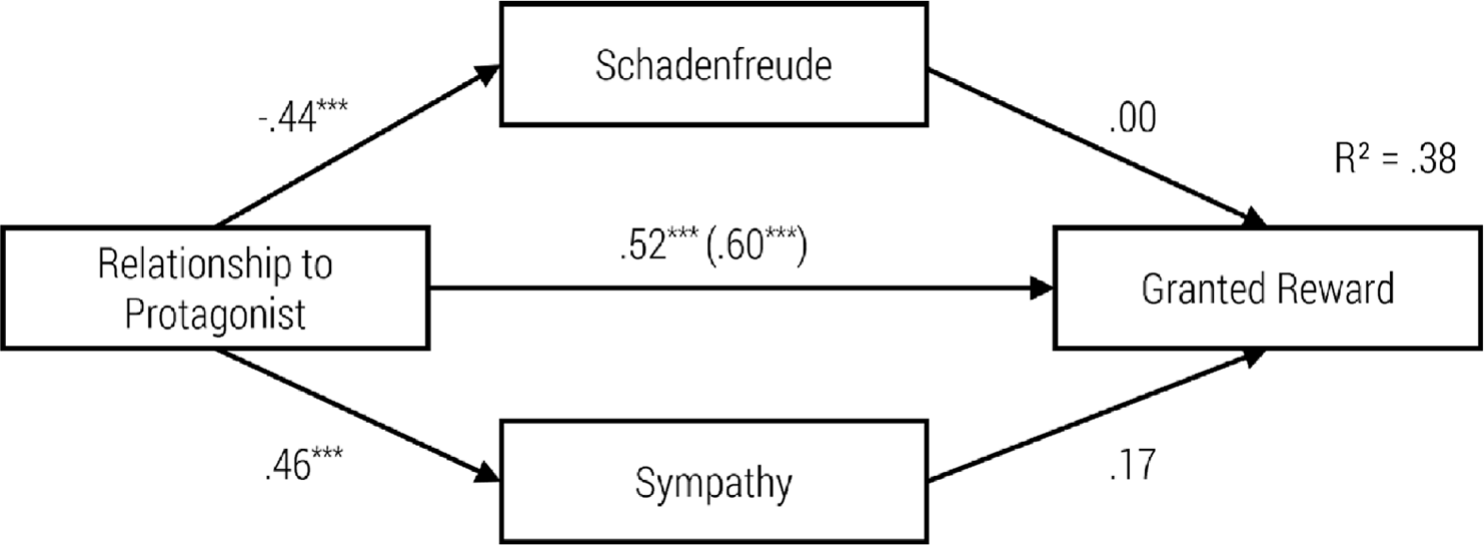
Path Model of the Impact of Relationship Type on Schadenfreude, Sympathy and Reward Granting with standardized regression coefficients β and the Impact without Schadenfreude and Sympathy in Brackets, ***p < .001.

To test the mediational role of the children’s feelings, we computed a path model with relationship type as a predictor, schadenfreude and sympathy as mediators, and reward granting as an outcome. Fig 8 shows the resulting model. We used bootstrapping procedures (10,000 trials) to overcome our small sample size.

There were significant direct predictions from relationship type to emotional reactions as well as to the behavioral measure. We also found an indirect effect of *r* = .08 from relationship type to the granted reward, when adding sympathy and schadenfreude as mediators between relationship type and the granted reward. This reduced the standardized coefficient for the direct path from r = .60 to r = .52. Nevertheless, the two emotional reactions could not predict to the behavioral measure significantly. Hence, due to the non-significant indirect paths the model did not confirm the mediation effect of schadenfreude and sympathy.

### Discussion

In this study, we focused on two research questions. First, we wanted to know whether one’s relationship to a protagonist (who fails to pursue an immoral goal) determines the experience of schadenfreude and sympathy. Furthermore, we hypothesized that these two emotions would predict actual prosocial behavior (i.e., reward granting) towards the unfortunate protagonist. A strength of our study is the realistic experimental manipulation we used. Unlike other studies dealing with fictional characters [33], our protagonists were real classmates of the children. Overall, children clearly displayed emotions, as can be seen in the absolute values of the moral emotional reactions to the picture stories. This clearly indicates that children even at young ages view life through the prism of morality. Even first grade students can formulate moral judgments and react on a spontaneous, fast, and efficient level. Moreover, the relationship with the actor had strong effects on feelings of sympathy and schadenfreude. This process seems to function as a simple heuristic [34] including the cognitive concept of like and dislike [12].

Our assumptions concerning the meditational role of sympathy and schadenfreude with respect to actual behavior, however, were not supported. Although the emotional reactions significantly predicted compensatory behavior, this effect disappeared when controlling for the children’s prior relationship with the protagonist. One explanation might be that compensatory behavior is not contingent upon the motivational triggering of emotional variables. For example, among young children, friendship expectations are dependent upon common activities and concrete long-term reciprocities rather than situational variables. Consistent with this understanding, Birch and Billman [35] found that only long-term friends share food with one another (and that this behavior cannot be generalized to children who are not friends).

A limitation of this study is that we restricted our analyses to immoral goals and negative intentions of the actor. In future studies, the pursuit of positive goals should be analyzed as well. Finally, an improvement in measuring actual behavioral choices seems appropriate. That is, the mere exhibit of schadenfreude might already represent a punishment to the actor. We could not test this assumption in our design. One potential method to analyze this is to introduce two possible behavioral reactions at the same time. For example actions of balancing, as well as actions of punishing the actor.

In sum, the personal relationship with a protagonist determines the amount of schadenfreude and sympathy children by the age of seven experience. In addition, both emotions predict the granting of a reward. However, we did not find a significant emotional mediation of behavioral choices.

## Study 3: Valence of Behavior, Responsibility and Granted Benefit

In Study 3, we examine the impact of attainment of a moral or immoral goal on schadenfreude and sympathy. For example, imagine that during winter time, a child stumbles and falls into the snow. Prior to this accident, this child had either helped another child, or had thrown a snowball at someone’s face. How do we and how do children react?

On one hand, by experiencing and expressing sympathy, we might provide a positive signal. On the other hand, by experiencing and expressing schadenfreude, we might send a negative signal. We hypothesize that sympathy is more likely when the observed child had previously been helpful to another child, while schadenfreude is more likely when the observed child had behaved aggressively.

In addition, we tested the effects of responsibility for a misfortune on the emotional reaction of an observer, by comparing a deliberately acting child who is controlling what happens with a child experiencing an uncontrollable incident caused by external forces [2]. If the child in the example jumped up and down despite the slippery snow and consequently fell, the child would be responsible for his tumble. An observer might display schadenfreude indicating that the child should be more careful. In contrast, if someone pushed the child, an observer would show sympathy as a way to show support. In summary, we hypothesize that both, negative behavior prior to one’s misfortune and high responsibility for one’s misfortune increases schadenfreude and reduces sympathy. Furthermore, we examine prosocial behavior more closely by analyzing how much the children would help emotional targets.

### Method

#### Participants

The managers and caregivers at 14 kindergartens were informed about the study in personal meetings. Parents were informed at parents’ evenings. After obtaining written consents, 157 children were interviewed. We excluded 15 children from the statistical analyses due to missing answers and/or poor comprehension of the stories. The final sample included *N* = 142 children (*M* = 74.15 months, *SD* = 12.18; 65 girls, 77 boys). We divided the children into four age groups for some of the following analyses: 14% were four years old (*M* = 54.15 months, *SD* = 3.68; 10 girls, 10 boys), 30% were five years old (*M* = 66.98 months, *SD* = 3.27; 23 girls, 20 boys), 28% were six years old (*M* = 77.35 months, *SD* = 3.40; 16 girls, 24 boys), and 28% were seven years old (*M* = 89.03 months, *SD* = 3.79; 16 girls, 23 boys).

#### Material and Procedure

As described previously, we designed a scenario-based interview. In a 2 x 2 x 2 within-subjects design, the picture stories systematically varied the gender of the protagonist (*male* vs. *female*), the valence of the behavior (*moral* or *immoral*) and the responsibility for the misfortune (*controllable* vs. *non-controllable*). This resulted in eight stories. We designed the stories and pictures carefully to control for potentially confounding factors. We used unusual names to avoid associations with existing children. As in the previous study, we trained the interviewer by test interviews that were videotaped and subsequently analyzed. Furthermore, we randomized the presented stories and questions to prevent sequence-effects.

**Fig 9:**
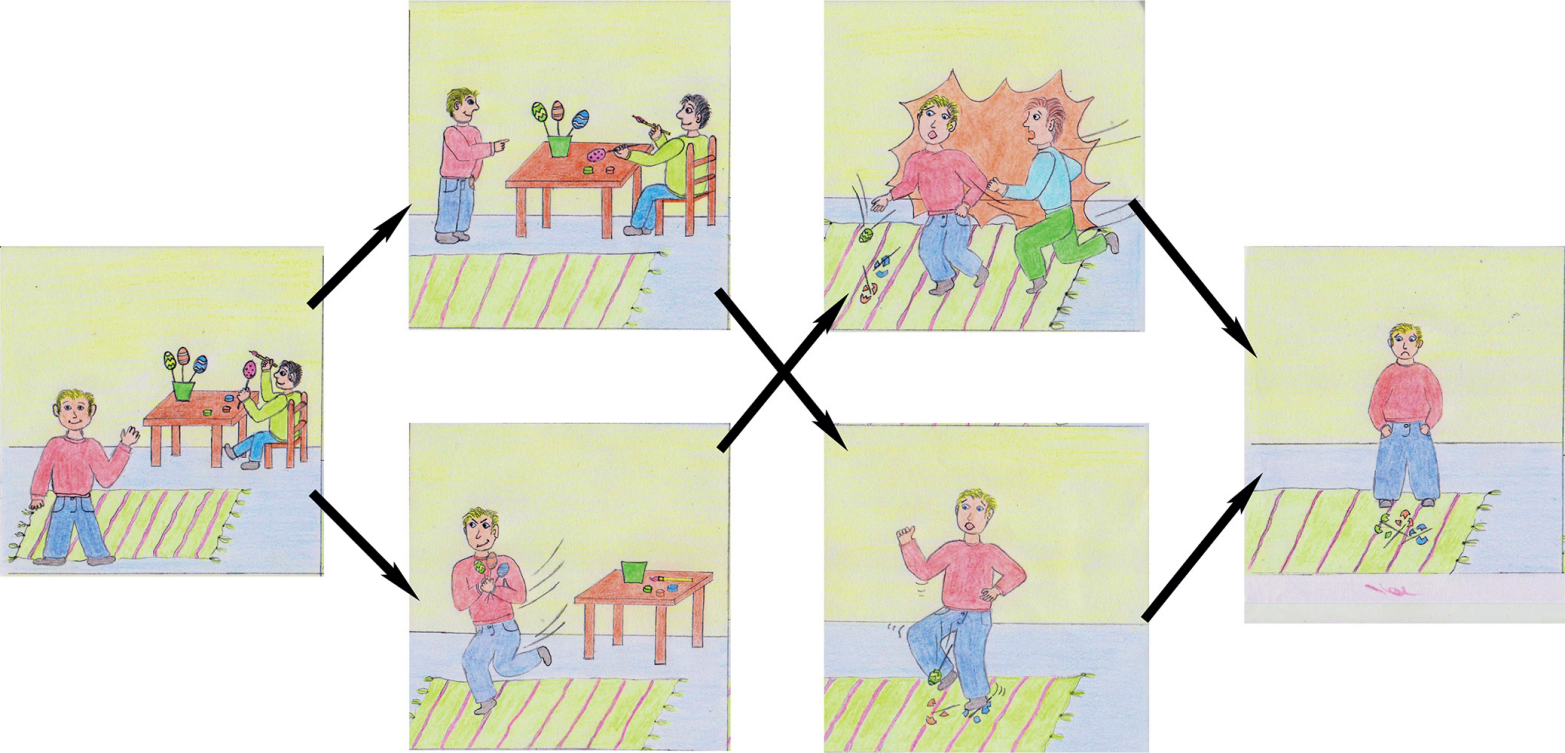
Example of picture story used in Study 3: “Ole and the Easter egg”.

Two female interviewers, who were about the same age, graduate students (psychology) and had both full knowledge of study hypotheses, tested the children. One of two female interviewers (person A or B) interviewed the children in a separate room at their respective institutions. Each session took about 15 minutes. We read the stories to the children while presenting the corresponding pictures presented in Fig 9. The scenario “Ole and the Easter egg” is about a boy called “Ole”. He is either stealing or asking for the beautiful Easter eggs which another child created. Subsequently, either another child pushes him and the eggs fall down and crack, or he drops the eggs (so they crack) because he is careless. In the scenario “Elfi and the Christmas star”, a girl called “Elfi” is either stealing or asking for the beautiful Christmas star, which another child created. Subsequently, either another child pushes her and the star falls down and breaks apart or she drops the star (so it breaks) because she is careless.

Afterwards, the children answered (in a random order) how much they experienced sympathy or schadenfreude, and the likelihood of helping the protagonist. In addition, they evaluated the severity of the protagonist’s misfortune and their willingness to grant him or her a reward. The children used a 5-point rating scale (0 = *not at all*, 4 = *very much*). The rating scale was displayed as a triangle and the children pointed to the section that matched their feelings. As in Study 2, we trained the children on how to use the scale. As in the previous study, all children received a small bag of sweets from the interviewer at the beginning of the interview. After each interview, the interviewer assessed the children’s comprehension of the stories, their comprehension of the scale, attention and motivation by using a 5-point rating scale (0 = *very poor*, 4 = *very good*).

### Results

We tested the influence of (1) gender of protagonist, (2) valence of behavior and (3) responsibility for misfortune on the dependent variables by using regression analyses. To control for confounding variables, we also included age and gender of the interviewed child, as well as the interviewer. For a clear interpretation of possible age effects we used contrast analyses.

Table 5 shows the mean values of the children’s emotional reactions (sympathy, schadenfreude), their behavioral tendencies (helping behavior, granting a reward), and perception of the severity of the misfortune, depending on the age of the children.

**Table 5:** Mean Values of Schadenfreude, Sympathy, Helping behavior, Granting of a Benefit and Severity of a Misfortune.

	4 Years	5 Years	6 Years	7 Years
	*M (SD)*	*M (SD)*	*M (SD)*	*M (SD)*
Schadenfreude	2.93 (1.85)	2.34 (1.48)	2.13 (1.57)	2.26 (1.39)
Sympathy	2.78 (1.67)	3.17 (1.47)	3.44 (1.55)	3.28 (1.50)
Helping behavior	3.70 (1.60)	3.93 (1.37)	3.73 (1.48)	3.33 (1.57)
Granting of a Reward	3.80 (1.49)	3.10 (1.74)	2.93 (1.85)	2.58 (1.92)
Severity of misfortune	3.25 (1.43)	3.50 (1.43)	3.63 (1.45)	3.51 (1.10)

#### Covariate Variables

**Participation quality:** The interviewer evaluated participants’ comprehension of the stories (*M* = 3.37, *SD* = 0.78), comprehension of the triangle rating scale (*M* = 3.15, *SD* = 1.00), attention and motivation (*M* = 3.48, *SD* = 0.68). Participants were evaluated as performing between good and very good in these domains.

**Severity of misfortune:** Children did not perceive the misfortunes in the stories as too severe overall (*M* = 3.47, *SD* = 1.35). Notably, severity was perceived as being higher in the first study. In this case, none of the proposed covariates showed a significant effect in the regression analysis.

#### Emotional Reactions

**Schadenfreude:** Valence of the behavior, responsibility and age influenced the experience of schadenfreude. Children reported more schadenfreude when the protagonist had stolen something, as when he had asked for something (see Table 6). Likewise, children indicated more schadenfreude when the protagonist was responsible as compared to when he was not responsible for his misfortune. In addition, for age of the children, a contrast analysis showed a significant linear effect, *F*(1, 284) = 5.24, *p* < .05, *R* ^2^ = .19, indicating that younger children reported more schadenfreude than older children. In addition, children reported more schadenfreude towards “Ole” than towards “Elfi”. Furthermore, there was a significant interviewer effect on the experience of schadenfreude, indicating that children reported more schadenfreude when interviewer A conducted the experiment. Gender of the children and the severity of the misfortune did not have significant effects.

**Table 6:** Multiple Regression for Schadenfreude and Empathy.

	Schadenfreude			Sympathy		
	*B*	*SE B*	β	*B*	*SE B*	β
Constant	2.38	0.64		-0.99	0.67	
Gender of interviewed Children	0.09	0.16	0.03	0.02	0.17	0.01
Age	-0.03	0.01	-0.22***	0.01	0.01	0.08
Interviewer	-0.81	0.16	-0.26***	0.22	0.17	0.07
Severity of Misfortune	0.09	0.06	0.08	0.27	0.06	0.24***
Gender of Protagonist	0.16	0.08	0.11*	0.08	0.08	0.05
Valence of Behavior	0.65	0.08	0.42***	-0.57	0.08	-0.37***
Responsibility	0.29	0.08	0.18***	-0.19	0.08	-0.12*

*Notes*.

Schadenfreude *R*² = .32

Sympathy *R*² = .23

*p < .05

***p < .001.

**Sympathy:** We found an effect for valence of behavior, such that children felt less sympathy and significantly more schadenfreude if the protagonist engaged in immoral behavior. There was also a significant effect of responsibility, as children felt more sympathy if the protagonist was not responsible for his misfortune (as compared to a responsible protagonist). In addition, children reported more sympathy when the misfortune was perceived as severe. None of the other proposed predictors had a significant effect.

#### Behavioral Tendencies

**Helping behavior:** Children helped the protagonist more frequently if the valence of the protagonist’s behavior was positive, i.e. when the protagonist had asked for something compared to stealing something (see Table 7). We obtained similar results for responsibility, such that the children preferred to help the protagonist if he or she was not responsible. In addition, an interviewer effect on helping behavior was observed, indicating that children reported more helping behavior when the experiment had been conducted by interviewer B. Gender of the protagonist, severity of the misfortune, age and gender of the interviewed children did not have significant effects.

**Table 7:** Multiple Regression for Helping Behavior and Granting a Reward.

	Helping Behavior			Granting a Reward		
	*B*	*SE B*	β	*B*	*SE B*	β
Constant	-0.34	0.61		3.06	0.84	
Gender of interviewed Children	0.12	0.15	0.04	-0.40	0.21	-0.09
Age	-0.01	0.01	-0.04	-0.04	0.01	-0.20***
Interviewer	0.83	0.15	0.28***	0.61	0.21	0.13**
Severity of Misfortune	0.02	0.06	0.01	-0.07	0.08	-0.04
Gender of Protagonist	0.10	0.07	0.07	-0.08	0.10	-0.04
Valence of Behavior	-0.69	0.07	-0.46***	-1.29	0.10	-0.57***
Responsibility	-0.29	0.07	-0.19***	-0.32	0.10	-0.14**

*Notes*.

Helping Behavior *R* ^2^ = .34

Granting a Reward *R* ^2^ = .44

**p < .01

***p < .001.

**Granting a reward:** Children were more likely to give the protagonist gummy bears if the protagonist’s behavior is moral in contrast to immoral behavior. There was a significant effect of responsibility, such that children gave more when a protagonist was not responsible for their misfortune. Furthermore, contrast analysis showed a significant linear effect of age, *F*(1, 284) = 7.40, *p* = .01, *R* ^2^ = .16, indicating that younger children granted more gummy bears than older children did. Moreover, there was a small interviewer effect on reward granting, indicating that children reported more helping behavior when interviewer B conducted the study. Gender of the protagonist, severity of the misfortune and gender of the interviewed children did not have significant effects.

**Fig 10:**
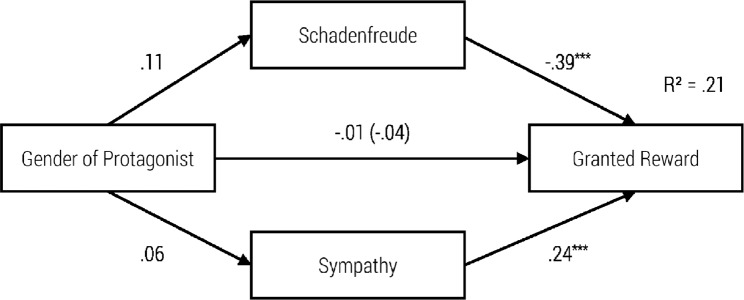
Path Model of the Impact of Gender of Protagonist on Schadenfreude, Sympathy and Reward Granting with standardized regression coefficients (β), ***p < .001.

**Fig 11:**
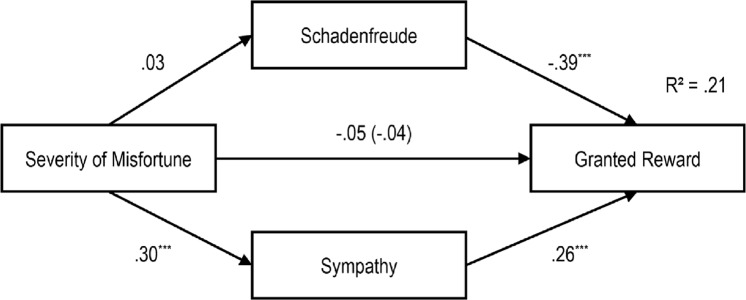
Path Model of the Impact of Severity of the Misfortune on Schadenfreude, Sympathy and Reward Granting with standardized regression coefficients β and the Impact without Schadenfreude and Sympathy in Brackets, ***p < .001.

**Fig 12:**
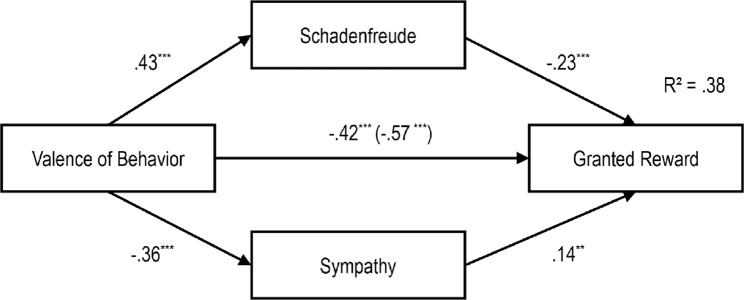
Path Model of the Impact of Valence of Behavior on Schadenfreude, Sympathy and Reward Granting with standardized regression coefficients β and the Impact without Schadenfreude and Sympathy in Brackets, **p < .01; ***p < .001.

**Fig 13:**
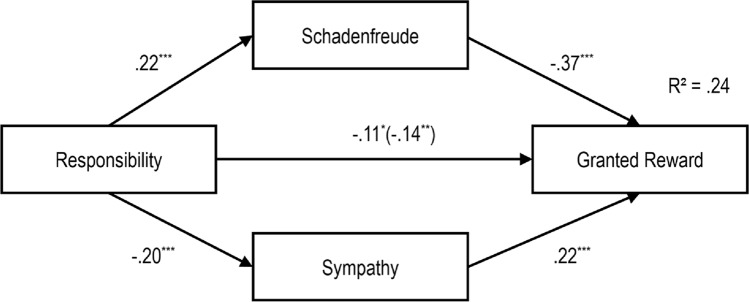
Path Model of the Impact of Responsibility on Schadenfreude, Sympathy and Reward Granting with standardized regression coefficients β and the Impact without Schadenfreude and Sympathy in Brackets, ***p < .001.

We integrated the above findings in four path models. We do so to illustrate the specific impact of our independent variables on schadenfreude, sympathy, and granting a reward (see Figs 10–13). We focus on granting a reward here, as it was an observed behavior enacted in our study. Nevertheless, similar patterns for intended helping behaviors were observed. With one exception, sympathy did not mediate the relation between protagonist’s gender and helping behavior

As can be seen in the first model (see Fig 10), the gender of our protagonist had no significant effect on schadenfreude. As can be seen, schadenfreude had a significant negative effect on granting a reward. Moreover, the gender of the protagonist had no significant direct influence on sympathy. However, sympathy had a significant positive effect on granting a reward. Nevertheless, the gender of the protagonist had no significant direct influence on the granting of a reward. Hence, due to the non-significant direct and indirect paths the model did not confirm the mediation effect of schadenfreude and sympathy.

In the second model (Fig 11), the severity of the misfortune had no significant effect on schadenfreude. As can be seen, schadenfreude had a significant negative effect on granting a reward. Nevertheless, the severity of the misfortune had a significant positive relationship with sympathy, and sympathy had a significant positive effect on granting a reward. However, the severity of the misfortune had no significant direct effect on the granting of a reward. Hence, due to the non-significant direct and indirect paths the model did not confirm the mediation effect of schadenfreude and sympathy.

In the third model (Fig 12), a positive significant effect of behavioral valence on schadenfreude, and a significant negative effect of schadenfreude on reward granting appeared. . . Furthermore, there was a significant negative relation between valence of behavior and sympathy, and a significant positive relation between sympathy and reward granting. The valence of behavior also had a direct significant negative effect on the number of gummy bears given to the protagonist. When Adding the indirect paths then the standardized coefficients decreased from *r* = -.57 to *r* = —.42Testing the whole model according to the procedure proposed by Hayes [25] indicated significant indirect effects for schadenfreude (z = -4.089, p > .000) and sympathy (z = -2.531, p = .011).

In the fourth model (Fig 13), responsibility had a significant positive effect on schadenfreude, and schadenfreude had a significant negative effect on reward granting. We also found an indirect effect of *r* = -.13 from responsibility to the reward granting, which resulted in a total effect of *r* = -.24 for this path. Furthermore, there was a significant negative relation between responsibility and sympathy, and a significant positive relation between sympathy and reward granting. Responsibility also had a direct significant negative effect on the number of gummy bears granted to the protagonist. When adding the indirect paths then the standardized coefficients decreased from *r* = —.14 to *r* = —.11. A mediation analysis was performed using the procedure proposed by Hayes [25] revealing significant indirect effects for schadenfreude (z = -3.380, p = .001) and sympathy (z = -2.711, p = .007).

### Discussion

In this study, we focused on two research questions. First, we wanted to know whether the valence of a behavior, the responsibility of a protagonist for a misfortune, and the severity of a misfortune, determines the experience of schadenfreude and sympathy. Furthermore, we hypothesized that these two emotions would predict enacted prosocial behavior (i.e., reward granting) towards the unfortunate protagonist.

#### Emotional Reactions

**Schadenfreude:** According to our results, schadenfreude is influenced by valence of the behavior, responsibility, age of the interviewed children, gender of the protagonist and the interviewer. Children were more likely to experience schadenfreude towards protagonists if they had behaved immorally prior to their misfortune than if they had behaved morally. This is consistent with the findings of Schulz et al. [23] that a morally bad goal of an protagonist prior to a misfortune elicits schadenfreude in an observer. In addition, our results corroborate previous findings that responsibility increases feelings of Schadenfreude [7].

In addition, younger children report more schadenfreude than older ones. As mentioned earlier, it is possible that children conceal schadenfreude due to social learning of display rules. Children also reported more schadenfreude towards “Ole” than to “Elfi”. This finding might be due to the gender of the protagonist or the differences in the story. The two stories differ in terms of the holiday (*Christmas* vs. *Easter*) and the presented handicrafts (*star* vs. *eggs*). Although the stories were designed to be very similar (both take place in the kindergarten and present the same misfortune), we cannot rule out that they were evaluated differently because of the small differences. Furthermore, despite great care we found an interviewer effect indicating that the two interviewers had a different effect on the amount of reported schadenfreude. Similar to the first study, one interviewer (person A) elicited a greater disclosure of schadenfreude in the children than the other.

**Sympathy:** Our results indicate that sympathy is determined by three factors: valence of the behavior, responsibility and severity of the misfortune. Children felt more sympathy when the protagonist was behaving morally prior to experiencing a misfortune. Moreover, children they felt less sympathy when the protagonist had engaged in an immoral act prior to the misfortune. Finally, sympathy increased when the protagonist was not responsible for his misfortune. This is consistent with Weiner’s [36] assumption that sympathy is more likely when an actor is not responsible for his misfortune. As expected, the severity of a misfortune is a particularly important determinant of his emotional reaction. Consequently, the more severe we judge an observed misfortune, the more sympathy we feel.

#### Behavioral Tendencies

**Helping behavior:** Since sympathy motivates helping behavior, the decision to help another child was, as expected, influenced by the valence of behavior and responsibility. In our study, an interviewer effect on helping behavior was also observed. Consistent with our findings on sympathy, helping behavior is increased when the protagonist behaved morally. Since helping is associated with potential costs to the helper, it is rational to invest resources only in persons who will likely return the favor see ([29]). People who have previously exhibited moral behavior are more likely to reciprocate altruism in the future. Helping behavior was also increased when the protagonist was not responsible for their misfortune. In addition, children reported more helping behavior to interviewer b than interviewer a did (see schadenfreude findings). The fact that mean helping behavior decreased with age is consistent with the results of Severy and Davis [37] who found that younger children (3–5 years) engaged in significantly more altruistic behavior than older children (8–10 years). This finding might be explained by the tendency for younger children to orient to standards set by adults and teenager to orient to peer norms.

**Granting of a Reward:** Consistent with our results regarding helping behavior, behavior valence, responsibility, age of the interviewed child, and the interviewer determined the amount of gummy bears granted. Children gave away more gummy bears to protagonists who were acting morally than protagonists acting immorally prior to their misfortunes. Furthermore, children tended to grant more gummy bears to protagonists who were not responsible for their misfortunes. In addition, younger children gave away more gummy bears than older children, which is in contrast to the findings of Fehr, Bernhard and Rockenbach [38] where the majority of three- and four-year-old children behaved selfishly and exhibited less egalitarianism. One potential explanation for the conflicting results is the fact that in our study, the gummy bears were “distributed” to the protagonist of a picture story, which was less realistic. Thus, it is possible that older children might be more likely to behave in socially desirable ways when they interact in everyday life situations. Finally, the children tended to give away more gummy bears when interviewed by one of the two interviewers (person B).

### Relations between Triggering Factors, Moral Emotions and Behavioral Tendencies

Guided by the results of the regression analyses, path models were developed. The models integrated schadenfreude and sympathy as mediators of the relation between gender of the protagonist, severity of the misfortune, valence of behavior and responsibility and reward granting.

Schadenfreude is not influenced by the protagonist’s gender and the severity of the misfortune; nevertheless, it has a direct impact on reward granting: Children were less likely to give rewards to protagonists when they felt schadenfreude. Likewise, sympathy is not influenced by the protagonist’s gender, however, it has a direct impact on reward granting. As hypothesized, the severity of a misfortune has an indirect effect on reward granting via sympathy. When children evaluated a misfortune as severe, sympathy, and consequently, the desire to grant a reward was increased. Likewise, children felt more sympathy towards, and granted more rewards to the female protagonist.

As expected, schadenfreude and sympathy mediate the effect of valence of behavior and responsibility on reward granting. When a behavior was evaluated as immoral, schadenfreude increased, and subsequently, fewer rewards were granted. Protagonists who were perceived as responsible for their misfortunes elicited more schadenfreude and were granted fewer rewards. Furthermore, children felt more sympathy and granted more rewards to protagonists who engaged in moral behavior. Lower perceived responsibility elicited more sympathy, which consequently, encouraged greater granting of rewards.

## Overall Discussion

### Summary

The presented studies provide insights into the causes and consequences as well as the development of schadenfreude and sympathy in children. In line with our expectations, children experienced sympathy as well as schadenfreude at the age of 4 years. Furthermore, children differentiated between these two emotions and their behavioral reactions towards another child’s misfortune. It becomes clear that children’s emotional and behavioral reactions are influenced by (1) whether the actor attained a goal prior to experiencing a misfortune, (2) their personal relationship with the actor, (3) the valence of a behavior prior to a misfortune, and (4) the actor’s responsibility for the experienced misfortune. Moreover, schadenfreude and sympathy appear as emotional opposites: Schadenfreude has a positive hedonic quality and at the same time represents a negative signal towards others, thus decreasing prosocial behavior. In contrast, sympathy has a negative hedonic quality, at the same time represents a positive signal towards others, thus increasing prosocial behavior.

#### Schadenfreude

Schadenfreude was more likely vis-à-vis misfortunes of disliked persons. Thus, children felt more schadenfreude for a suffering child they disliked as compared to a child they liked. In addition, children felt more schadenfreude for a child who pursued an immoral goal (as compared to a moral goal). Children also felt more schadenfreude towards an actor who attained a morally negative goal than someone who failed to attain this goal. Furthermore, observers felt more schadenfreude for actors who were responsible for their misfortunes than for those whose misfortunes were perceived as uncontrollable. Finally, schadenfreude promotes avoidance tendencies towards the emotional target, as indicated by a decreased tendency to help, to sit next to someone, or to grant a reward.

#### Sympathy

Sympathy was more likely vis-à-vis misfortunes of liked persons. Thus, children felt more sympathy for a suffering child they liked as compared to a child they disliked. In addition, children felt more sympathy for a child who pursued a moral goal (as compared to an immoral goal). Children also felt more sympathy for someone who failed to attain an immoral goal than someone who attained such a goal. Furthermore, observers felt more sympathy for actors whose misfortunes were perceived as uncontrollable (as compared to actors who were responsible for their misfortunes). Finally, sympathy promotes approach tendencies towards the emotional target, as indicated by an increased tendency to help, to sit next to someone, or to grant a reward.

### Limitations and Future Perspectives

We would like to address two methodological issues, which represent severe obstacles that need to be overcome in future research in this field (1) the suggestibility of preschool children, (2) the limitations of our scenarios due to the differences in content combined with gender differences of the featured protagonist and the limitations of closed questions.

#### Suggestibility

Despite careful interviewer training, our interviewers might have influenced the interviewed children by way of non-verbal gestures and intonations. Generally, preschool children are vulnerable to suggestion [39,40]. Since children tend to be cooperative interaction partners, they supply others with the type of information they think is being requested [41]. Future studies could attempt to conceal the speaker's intent by using a cover story and thus avoid that children give answers they think the interviewer anticipates.

#### Scenario differences and gender effects

A limitation of Study 1 and 3 was the link between the scenarios (actions) and the protagonist’s gender. In both studies, we cannot be certain whether the differences in response emotion (sympathy and schadenfreude) were the result of the differences in the stories or gender. In the first study, the setting, actions and the misfortunes were different for the two stories. Thus, an interpretation of the observed story effects is more difficult. Therefore, we designed the scenarios for the Study 3 in a much more similar way. Nevertheless, we cannot rule out that the children judged them differently because of small differences (e.g., differences between Christmas and Easter, or time of the year, and so on). On the other hand, the gender of the protagonist might have influenced the judgments of the children. It is commonly acknowledged that gender stereotypes serve to organize thought and guide action [42]. Consequently, it might affect children's evaluative judgments of other persons [43]. It is possible that children tend to react differently to the stories due to their differing attitudes towards female and male protagonists. Thus, the statements of the children might have reflected the belief that it is more acceptable to feel more sympathy and less schadenfreude for girls than boys. Consequently, further studies are needed to analyze potential gender or scenario effects.

#### Qualitative Data

Due to the specific questionnaire and answer format and the quantitative nature of our data, our insights concerning the actual experience of schadenfreude and sympathy are certainly restricted to this kind of methodology. In addition, we regard the collection of qualitative data as a highly useful strategy for future studies. This strategy might be especially advisable for older subsamples (likely beginning at the age of six years).

### Implications

The presented studies shed light on the determinants of schadenfreude and sympathy in children as well as on subsequent behavioral reactions. Schadenfreude and sympathy as moral emotions are crucial elements of social skills, as they determine the success of social interactions [44]. Future studies should focus on effort as a predictor of schadenfreude and sympathy, as results from research with adults indicates that effort might be another important determinant of emotional responding [11]. These studies would complement the findings of the present study nicely since effort is a one of several key components of goal attainment, along with controllability and responsibility [2], which were investigated here.
